# Stereotactic body radiation therapy using a respiratory-gated volumetric-modulated arc therapy technique for small hepatocellular carcinoma

**DOI:** 10.1186/s12885-018-4340-7

**Published:** 2018-04-13

**Authors:** Yuri Jeong, Jinhong Jung, Byungchul Cho, Jungwon Kwak, Chiyoung Jeong, Jong Hoon Kim, Jin-hong Park, So Yeon Kim, Ju Hyun Shim, Kang Mo Kim, Young-Suk Lim, Han Chu Lee, Sang Min Yoon

**Affiliations:** 1 Department of Radiation Oncology, Asan Liver Center, Asan Medical Center, University of Ulsan College of Medicine, 88, Olympic-ro 43-gil, Songpa-gu, Seoul, Republic of Korea; 2 Department of Radiology, Asan Liver Center, Asan Medical Center, University of Ulsan College of Medicine, Seoul, Republic of Korea; 3 Department of Gastroenterology, Asan Liver Center, Asan Medical Center, University of Ulsan College of Medicine, Seoul, Republic of Korea

**Keywords:** Hepatocellular carcinoma, Local control rate, Stereotactic body radiation therapy, Volumetric-modulated arc therapy, Overall survival

## Abstract

**Background:**

Volumetric-modulated arc therapy (VMAT) is a highly sophisticated linear accelerator-based treatment method, and allows dose rate-changing intensity modulation with gantry rotation. We report our clinical experiences with stereotactic body radiation therapy (SBRT) using a respiratory-gated VMAT technique for patients with hepatocellular carcinoma (HCC) when established curative treatments cannot be applied.

**Methods:**

A total of 119 patients (139 lesions) with HCC who were treated with SBRT were registered between March 2012 and July 2013 at our institution. A dose of 10–15 Gy per fraction was applied over 3–4 consecutive days, resulting in a total dose of 30–60 Gy.

**Results:**

The median follow-up period was 25.8 months (range, 3.2–36.8 months). The overall 3-year survival rate was 83.8%. The local control rate at 3 years was 97.0% in all treated lesions. Multivariate analysis revealed that the Child-Pugh class before SBRT had significant effects on overall survival (Child-Pugh A: hazard ratio = 0.463; 95% CI, 0.262–0.817; *p* = 0.008).

**Conclusions:**

SBRT using a respiratory-gated VMAT technique was an excellent ablative treatment modality for patients with HCC. SBRT is a good alternative treatment for patients with small HCCs that are unsuitable for surgical resection or local ablative therapy.

**Electronic supplementary material:**

The online version of this article (10.1186/s12885-018-4340-7) contains supplementary material, which is available to authorized users.

## Background

Hepatic resection, liver transplantation, and radiofrequency ablation (RFA) are recommended as curative treatment options for hepatocellular carcinoma (HCC) [1]. However, only 10 to 30% of patients are suitable for hepatic resection for various clinical reasons [2]. In patients with small HCC and preserved liver function, RFA provided an excellent local control rate and favourable survival rate [1, 3, 4]. According to several randomized trials comparing RFA with hepatic resection as a first-line therapy for small HCC, survival rates after RFA were similar to those after hepatic resection, even though all trials did not reach the same conclusions [5–7]. However, the use of RFA is limited when the tumors are undetectable by ultrasonography or positioned at the surface of the liver, at the top of the dome, or near the bile duct or large vessels.

With recent advances in the radiotherapy techniques, stereotactic body radiation therapy (SBRT) has been considered an alternative treatment option for small HCC that is not suitable for hepatic resection and RFA [8, 9]. Based on phase I/II studies that evaluated the feasibility and safety of SBRT in primary and metastatic liver tumors [10–12], several retrospective studies evaluated the local ablative role of SBRT for small HCC; these studies showed favorable local control and survival rates [13–16]. More recently, volumetric-modulated arc therapy (VMAT), one of the most sophisticated linear accelerator-based treatment modalities, has become available even with gated delivery. VMAT allows dose rate-changing intensity modulation with gantry rotation, and may provide more conformal dose distribution while reducing treatment delivery time and monitor units. In the present study, we report our clinical experiences with SBRT using a respiratory-gated VMAT technique as an alternative treatment for small HCC.

## Methods

### Patients

We retrospectively reviewed a registered database of patients who received SBRT using a respiratory-gated VMAT technique for primary or recurrent HCCs between March 2012 and July 2013. The inclusion criteria for the present study were the same as those for our previous study [16]. Patients were selected to receive SBRT for the following reasons: (1) surgery was contradicted because of liver cirrhosis, insufficient remnant liver for resection, or patient refusal to undergo surgery; (2) RFA was not applicable because of the location of HCC, undetectable HCC on ultrasonography, or the bleeding tendency of the patients; or (3) lesion non-visibility on hepatic angiogram for transarterial chemoembolization (TACE) or an incomplete response after TACE.

### Simulation and treatment planning

The simulation and target volume delineation procedures were the same as those described in our previous study [16]. All patients were immobilized with a vacuum cushion in the supine position. Free-breathing 4-dimensional (4D) computed tomography (CT) scanning was performed using a 16-slice CT system (GE LightSpeed RT 16; GE Healthcare, Waukesha, WI, USA). To analyse the patients’ breathing patterns, a Real-time Position Management respiratory gating system (Varian Medical Systems, Palo Alto, CA, USA) was used, and all CT datasets were sorted into 10-phase bins that corresponded to the respiratory phase, using 4D imaging software (Advantage 4D; GE Healthcare).

The respiratory-gated VMAT technique was delivered using a 10-MV flattening filter-free beam from a Varian TrueBeam STx (Varian Medical Systems) with multileaf collimators (MLCs) comprising 120 leaves of 2.5 mm width in sliding window mode. Two arc beams, the first arc beam of clockwise rotation and the second arc beam of counter-clockwise rotation, were used for the VMAT plan. The range of arc angle varied from 360° to < 180° according to the location of the target volume and the organs at risk (OARs), and the collimator angle was 30° for the first arc and 330° for the second arc. VMAT plans were optimized by Eclipse progressive resolution optimization (version 10.0.28, Varian Medical Systems)) and dose calculations were performed using an Anisotropic Analytic Algorithm, with a maximum dose rate of 1200 MU/min.

A total dose of 30–60 Gy (median: 45 Gy) was applied to the isodose line covering the planning target volume and a dose of 10–20 Gy (median: 15 Gy) per fraction was applied over 3–4 consecutive days. The total dose was mainly determined based on general dosing guidelines after estimating the dose to be administered to the normal liver and other critical OARs, according to our previous study [16].

Before each fraction of SBRT, image guidance for the fiducial markers that were located around the tumor was performed in 2 stages using an On-Board Imager (Varian Medical Systems), as described in our previous study [16]. At least 1 week before CT simulation, 3 gold seeds as the fiducial markers were implanted into the liver parenchyma around the tumor, using sonographic guidance. In patients who had surgical clips or compact iodised oil remaining after previous treatments, or who had HCC in the hepatic dome, gold seeds were not implanted. Instead, the surgical clips, compact iodised oil, or hepatic dome was used for image guidance.

### Evaluation and statistics

All patients were examined during SBRT, and regular follow-up examinations were performed at 2–3 month intervals after treatment. Adverse effects related to SBRT were graded according to the Common Terminology Criteria for Adverse Events (CTCAE; version 4.0). Radiation-induced liver disease (RILD) was defined as classic (anicteric hepatomegaly and ascites, or elevation of alkaline phosphatase to more than twice above the upper limit of normal or baseline level) or non-classic (elevation in the level of transaminases or bilirubin, which was graded according to CTCAE, or a decline in liver function measured by a worsening of Child-Pugh score ≥ 2) in the absence of disease progression within 3 months after SBRT [17]. Logistic regression analysis was performed to evaluate association of variables with RILD.

Radiologic response was evaluated by multiphase dynamic CT scans or magnetic resonance imaging according to the modified Response Evaluation Criteria in Solid Tumors (mRECIST) criteria [18]. Local failure, intrahepatic recurrence, and distant metastasis were defined as the recurrence of the treated lesion, recurrence within the liver but outside the treated lesion, and recurrent disease at any site outside the liver, respectively. Overall and recurrence-free survival rates were estimated from the date of the start of SBRT to the date of death or the last follow-up, and to the date of tumor recurrence or last follow-up, respectively, using the Kaplan-Meier method. Univariate and multivariate Cox proportional hazards models were generated to describe the association of variables with recurrence-free survival and overall survival. Backward elimination Cox’s regression was used to select the principal risk factors in the multivariate model, and variables with *p* values ≤0.2 in univariate analysis were chosen for multivariate analysis. Logistic regression analysis was performed to confirm the variable-effect relationships. These analyses were two-sided and performed at the 5% level of significance by using the SPSS software (version 21; IBM SPSS Statistics, Armonk, NY, USA).

## Results

### Patient characteristics

A total of 161 patients with HCC were treated with SBRT using respiratory-gated VMAT technique during the study period. Among them, 45 patients were excluded from our analysis for the following reasons: presence of major vascular invasion (*n* = 2), extrahepatic metastases (*n* = 6), Child-Pugh class C (*n* = 1), complete response after previous TACE (*n* = 5), prior history of radiotherapy to the liver (*n* = 8), loss to follow-up (*n* = 10), history of malignancy other than HCC (*n* = 2), and liver transplantation before or after SBRT (*n* = 8). The remaining 119 patients (139 lesions) met all of the enrolment criteria and were included in this analysis (Table 1). At the time of SBRT, only 3 patients (2.5%) were treatment-naïve, and all other patients had previously received various therapies, including hepatic resection, RFA, percutaneous ethanol injection (PEI), TACE, or sorafenib before SBRT. The median number of prior treatment sessions was 3 (range, 1–25 courses). The normal liver volume was median 1185.8 cm^3^ (range, 701.2–2102.2 cm^3^).

**Table 1:** Patient characteristics

Variables	No. of patients (%)
Sex	
Male	97 (81.5)
Female	22 (18.5)
Age (years)	
Median (range)	60 (36–90)
ECOG performance status	
0	102 (85.7)
1	13 (10.9)
2	4 (3.4)
Child-Pugh class	
A	108 (90.8)
B	11 (9.2)
Viral aetiology	
Hepatitis B virus	93 (78.2)
Hepatitis C virus	11 (9.2)
Non B, Non C	15 (12.6)
Tumor size (cm)^a^	
Median	1.7
0.8–1.0	8 (5.8)
1.1–2.0	90 (64.7)
2.1–3.0	32 (23.0)
3.1–4.0	8 (5.8)
5.1–6.0	1 (0.7)
Modified UICC stage^b^	
I	60 (50.4)
II	53 (44.5)
III	6 (5.0)
Alpha-fetoprotein (ng/mL)	
Range	1.6–4303.6
PIVKA-II (mAU/mL)	
Range	0–11,501.0
No. of prior treatment before SBRT	
Median (Range)	3 (0–25)
Summary of prior treatments	
None	3 (2.5)
TACE	57 (47.9)
TACE, RFA	27 (22.7)
TACE, PEI	3 (2.5)
TACE, RFA, PEI	1 (0.8)
TACE, sorafenib	1 (0.8)
Resection	2 (1.7)
Resection, TACE	15 (12.6)
Resection, TACE, RFA	5 (4.2)
Resection, TACE, PEI	2 (1.7)
Resection, RFA	2 (1.7)
PEI	1 (0.8)

*ECOG* Eastern Cooperative Oncology Group, *UICC* Union for International Cancer Control, *PIVKA-II* prothrombin induced by vitamin K absence-II, *SBRT* stereotactic body radiation therapy, *TACE* transarterial chemoembolization, *RFA* radiofrequency ablation, *PEI* percutaneous ethanol injection

^a^One hundred and thirty-nine tumors were analysed for size

^b^Modified UICC stage was assessed by the viable tumor at the timing of SBRT

### Radiologic response and recurrences after SBRT

**Fig. 1:**
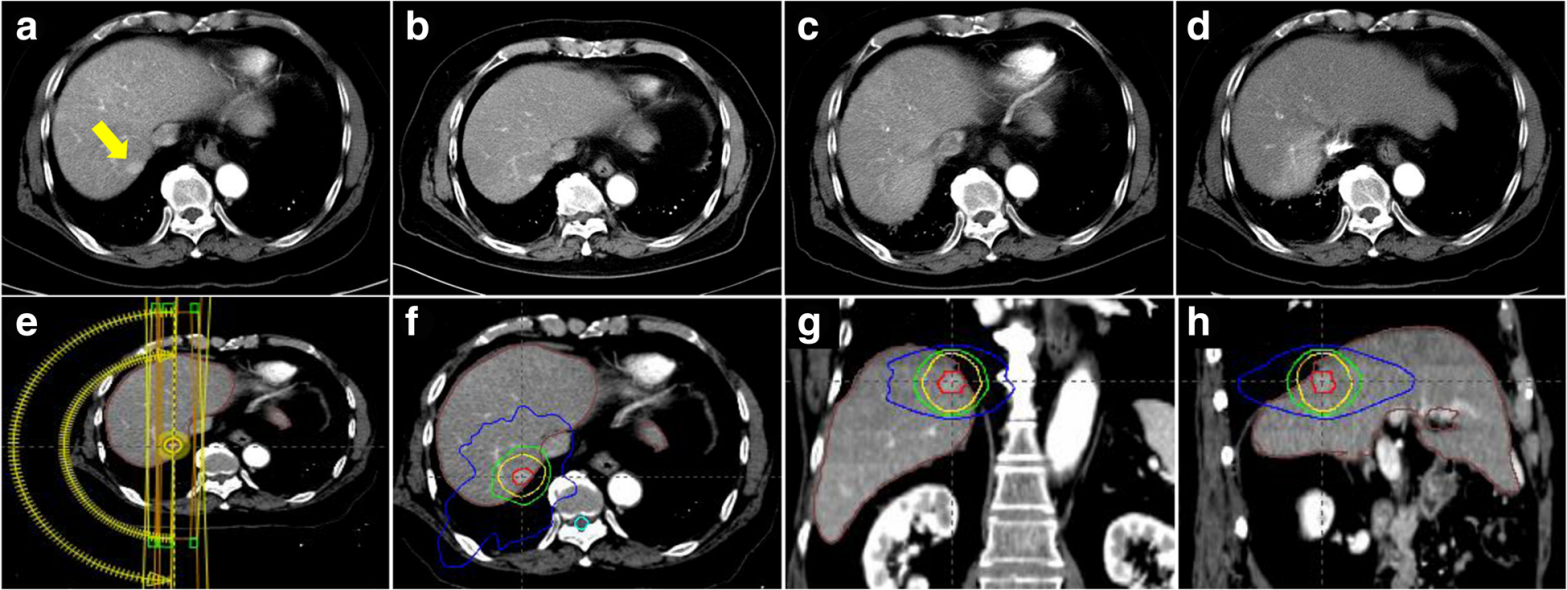
SBRT using a respiratory-gated VMAT technique. A representative patient with HCC (located in segment 7) who achieved complete response at 6 months after SBRT. CT scans in arterial phase before SBRT (**a**), 1 month (**b**), 3 months (**c**), and 6 months (**d**) after SBRT, respectively. CT scans for VMAT plan which shows 2 arc beams (**e**) and isodose lines in axial (**f**), coronal (**g**), and sagittal (**h**) views. The red line is the gross tumor volume, and yellow, green, and blue lines are the 97, 70, and 30% isodose lines, respectively

**Fig. 2:**
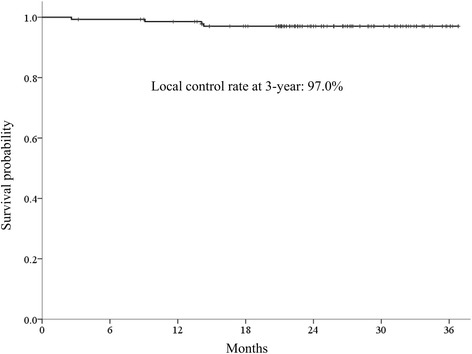
Local control rate in all treated lesions. Local control rates at 3 years was 97.0%

**Fig. 3:**
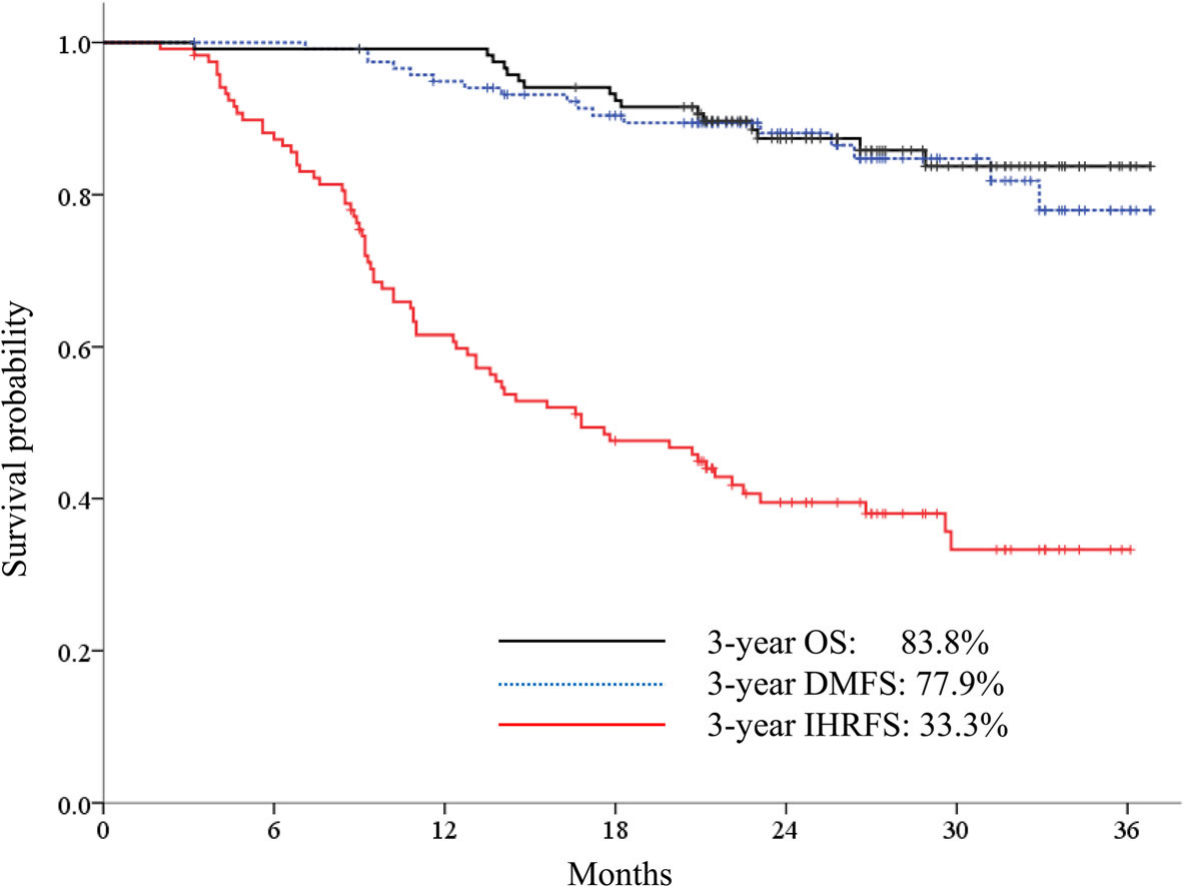
Survival outcomes. Overall survival, distant metastasis-free survival and intrahepatic recurrence-free survival rates in all patients

The median follow-up period for all patients was 25.8 months (range, 3.2–36.8 months). The radiologic responses at 6 months after completion of SBRT were available 137 (98.6%) of 139 lesions, and complete response, partial response, and stable disease were achieved in 124 (89.2%), 9 (6.5%), and 4 (2.9%) patients, respectively. Progressive disease was not observed at the timing of the response evaluation. Figure 1 shows a representative patient who achieved complete response at 6 months after SBRT. Local control rates at 1 and 3 years were 98.5 and 97.0%, respectively (Fig. 2). Four patients who experienced local failure also developed intrahepatic recurrence and/or distant metastasis. Distant metastasis-free survival rates at 1 and 3 years were 94.9 and 77.9%, respectively (Fig. 3). Intrahepatic recurrence was the major pattern of failure (72 of 119 patients) and intrahepatic recurrence-free survival rates (IH-RFS) at 1 and 3 years were 61.5 and 33.3%, respectively (Fig. 3). In multivariate analysis, the prior treatment session (Hazard ratio (HR) = 1.087; 95% confidence interval (CI), 1.031–1.146; *p* < 0.001) and age (HR = 1.025; 95% CI, 1.001–1.049; *p* = 0.039) were significant factors for IH-RFS (Table 2).

**Table 2:** Factors influencing intrahepatic recurrence-free survival and overall survival after stereotactic body radiotherapy

	Intrahepatic recurrence-free survival rates				Overall survival rates			
	Univariate analysis		Multivariate analysis		Univariate analysis		Multivariate analysis	
Variables	HR (95% CI)	*p* value	HR (95% CI)	*p* value	HR (95% CI)	*p* value	HR (95% CI)	*p* value
Gender (male)	0.879 (0.490–1.577)	0.666			0.531 (0.184–1.529)	0.241		
Age	1.021 (0.998–1.045)	0.076	1.025 (1.001–1.049)	0.039	1.024 (0.977–1.074)	0.319		
ECOG performance status (0)^a^	0.751 (0.403–1.397)	0.365			0.457 (0.147–1.420)	0.176	–	–
Child-Pugh class (A)	1.102 (0.699–1.737)	0.677			0.458 (0.259–0.808)	0.007	0.463 (0.262–0.817)	0.008
Viral aetiology (HBV)^b^	0.928 (0.459–1.877)	0.836			0.440 (0.140–1.382)	0.160	–	–
Tumor size	1.006 (0.726–1.393)	0.971			0.863 (0.385–1.938)	0.721		
Alpha-fetoprotein	1.000 (1.000–1.001)	0.628			0.998 (0.990–1.006)	0.664		
PIVKA-II	1.000 (1.000–1.000)	0.448			0.999 (0.995–1.003)	0.712		
No. of prior treatment sessions	1.076 (1.024–1.131)	0.004	1.087 (1.031–1.146)	0.002	0.891 (0.735–1.080)	0.238		

*HR* hazard ratio, *CI* confidence interval, *ECOG* Eastern Cooperative Oncology Group, *HBV* hepatitis B virus, *PIVKA-II* prothrombin induced by vitamin K absence-II

^a^Categorical variables are divided as ECOG performance status 0 vs. 1–2

^b^Categorical variables are divided as HBV (+) vs. HBV (−)

### Additional treatment after SBRT for new recurrent lesions

For new recurrent lesions, most patients (73 of 77 patients who experienced recurrences) received various additional treatments, including locoregional treatments for intrahepatic recurrences (e.g., segmentectomy, RFA, PEI, TACE, SBRT, or conventional radiotherapy) or treatments for distant metastases (e.g., sorafenib, metastasectomy, SBRT, or palliative radiotherapy).

### Overall survival rates

**Fig. 4:**
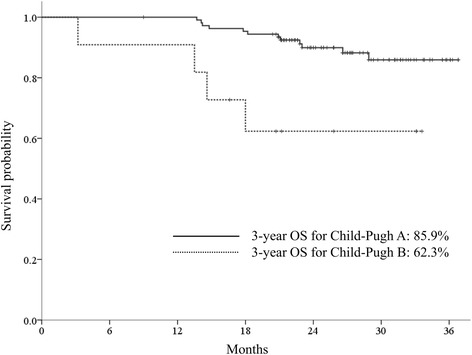
Overall survival rates according to the Child-Pugh class. The 3-year overall survival rates were 85.9 and 62.3% in patients with Child-Pugh class A and Child-Pugh class B, respectively (*p* = 0.003)

At the time of analysis, 103 patients were alive and 16 patients had died. The 1- and 3-year overall survival rates were 99.2 and 83.8%, respectively (Fig. 3). In the multivariate analysis, Child-Pugh class before SBRT was the only significant prognostic factor for overall survival (Child-Pugh A: HR = 0.463; 95% CI, 0.262–0.817; *p* = 0.008) (Table 2). The 3-year overall survival rates were 85.9 and 62.3% in patients with Child-Pugh class A and Child-Pugh class B, respectively (*p* = 0.003) (Fig. 4).

### Treatment-related toxicity

Treatment-related toxicities are summarized in Table 3. All patients received the planned SBRT without any interruptions, and no patient experienced classic RILD. Elevation in the level of transaminases or bilirubin of CTCAE grade ≥ 2 that may have been related to SBRT without progression of intrahepatic HCC were observed in 10 (8.4%) patients. The worsening of Child-Pugh score ≥ 2 were observed in 7 patients with the elevation in the level of transaminases or bilirubin of CTCAE grade 1 (*n* = 2), grade 2 (*n* = 4), and grade 3 (*n* = 1), respectively. In the logistic regression analysis, no factor was associated with RILDs which was defined as the elevation in the level of transaminases or bilirubin of CTCAE grade ≥ 2 or worsening of Child-Pugh score ≥ 2.

**Table 3:** Acute and late toxicities after stereotactic body radiotherapy for hepatocellular carcinoma

		No. of patients (%)				
	Adverse events	Grade 1	Grade 2	Grade 3	Grade 4	Grade 5
Acute	Non-classic RILD	38 (31.9)	8 (6.7)	2 (1.7)	–	–
	Fatigue	12 (10.1)	–	–	–	–
	Anorexia	2 (1.7)	–	–	–	–
	Nausea	8 (6.7)	–	–	–	–
	Pain	2 (1.7)	1 (0.8)	–	–	–
	Diarrhoea	–	–	–	–	–
Late	Fracture (rib)	7 (5.9)	2 (1.7)	–	–	–
	Pneumonitis	20 (16.8)	–	–	–	–
	Stricture (biliary tract)	5 (4.2)	–	2 (1.7)	–	–

*RILD* radiation-induced liver disease

The most common acute toxicities other than hepatic toxicities were grade 1 fatigue (10.1%) and grade 1 nausea (6.7%). During the follow-up period, 9 (7.6%) patients developed rib fractures between 10 and 30 months after SBRT for peripheral tumors that were located near the ribs; however, most of these patients did not require any specific treatment. Grade 3 biliary strictures that required endoscopic intervention occurred in 2 (1.7%) patients at 12 and 20 months after SBRT for central lesions, respectively. Other gastrointestinal toxicities such as bleeding or perforation were not observed.

## Discussion

In the present study, 119 patients (139 lesions) with small, primary, or recurrent HCC were treated with SBRT using a respiratory-gated VMAT technique. Although more than half of patients experienced intrahepatic recurrences outside the treated lesions, the treated lesions themselves were well controlled and showed an excellent local control rate of 97% at 3 years. In addition, overall survival rates were good (83.8% at 3 years).

The local control rate described in the present study seemed to be similar to that of RFA; this suggested the possibility of SBRT as an ablative therapy for small HCC, even though most of the lesions described in the present study were recurrent cases after various courses of previous therapies, and therefore might have behaved more aggressively. In a study of RFA, the local recurrence rates were reported from 3.2 to 18% at 3 years for early stage HCC by using the Milan criteria, and less than 3% for the solitary, smaller HCC (≤2 cm) with liver function of Child-Pugh class A [4, 19, 20]. There are 3 possible reasons for the excellent local control rate in the present study. Firstly, the median size of tumors was as small as 1.7 cm, and most tumors (93.5%) were less than 3 cm. In our previous study, which analysed 93 patients treated with SBRT, the local control rate was 92.1% at 3 years and tumor size was a significant prognostic factor for local control rates [16]. Tumor burden, including tumor size and/or volume, was reported as a significant prognostic factor for local control rates in several other studies of RFA [3, 19, 21] and SBRT [22, 23]. Secondly, our target volume definition, dose prescription, and treatment delivery methods might be appropriate for the ablation of small (less than 3 cm) HCCs, even though the optimal details of SBRT for certain sizes of HCC have not yet been defined. Finally, respiratory-gated VMAT can shorten the treatment delivery time compared with SBRT using static beams. Although the clinical impact of prolonged treatment delivery time on the SBRT at a high dose per fraction is not yet clear, from a biological perspective, a prolonged delivery time has shown a detrimental effect on tumor control by reducing cell killing in cell lines or in xenograft models of tumors with a low alpha/beta ratio [24, 25].

The major pattern of failure was intrahepatic recurrence and IH-RFS were as low as 33.3% at 3 years. Intrahepatic recurrence was also reported as a major pattern of failure in many other studies of hepatic resection [5] or RFA [3, 5, 19–21], as well as SBRT [14, 16, 23, 26]. Intrahepatic recurrence accounted for 78 to 96% of overall recurrence that occurred after hepatic resection [27], and ranged from 45 to 52% at 3 years and more than 70% at 5 years after RFA [3, 19–21]. Intrahepatic recurrences are thought to result from multicentric carcinogenesis, which is an underlying risk of HCC, as well as intrahepatic metastasis from the primary HCC. Considering that local treatments cannot prevent the underlying risk of HCC development, intrahepatic recurrences seem to be inevitable. In the present study, the number of prior treatment sessions was the only significant prognostic factor for IH-RFS and it seems to be associated with factors. To reduce intrahepatic recurrences, further studies investigating the combination of regional or systemic therapies in SBRT are needed, especially when treating patients with recurrent HCC who have undergone multiple prior treatments.

In the present study, the overall survival rate was 83.8% at 3 years. Although direct comparisons are difficult because of differences in patient, tumor, and treatment characteristics, the survival rate of the present study seems to be higher than that of any other SBRT studies; there may be several reasons for this. Firstly, most patients had preserved liver function. In the present study, Child-Pugh class before SBRT was the only prognostic factor for overall survival When compared with our previous report, in which the 3-year overall survival rate was 53.8%, the present study included more patients with liver function of Child-Pugh class A (90.8% vs. 74.2%) [16]. Secondly, the excellent local control rate found in the present study may be associated with improved survival. In several studies regarding RFA and SBRT, the local complete response and/or local recurrence were significant prognostic factors for overall survival [22, 28, 29]. In a study by Takahashi et al., in which RFA was applied to patients with early stage HCC and liver function of Child-Pugh class A, local recurrence was a significant prognostic factor for survival (5-year overall survival: 84.4% vs. 42.1% (*p* = 0.0002) [29]. Thirdly, aggressive treatments for new recurrent lesions might contribute to improved survival rates. In the present study, most patients received additional treatments for new recurrent lesions. Of the 32 patients who received local ablative therapies for intrahepatic recurrence, only 1 patient died of disease progression.

This study has also limitations because of its retrospective nature and short follow-up period. To validate the excellent local control and favourable survival rates of the present study, further prospective investigation with long-term follow-up is needed. Recently, our institution has completed enrolment of patients in a phase II prospective study to evaluate the efficacy of SBRT for unresectable HCC (CRIS registration number: KCT00000625); this study can provide information about the role of SBRT as an ablative treatment option for HCC.

## Conclusions

SBRT using a respiratory-gated VMAT technique was an excellent ablative treatment modality for patients with small HCC. SBRT can be a good alternative treatment for patients with small HCCs that are unsuitable for surgical resection or local ablative therapy.

## Additional files

Additional file 1: Raw data of the patients. Patient characteristics and follow-up data exists. (XLSX 24 kb)

Additional file 2: Raw data of the treated lesions. Lesion characteristics and follow-up data exists. (XLSX 17 kb)

## Abbreviations

4D: 4-dimensional
CI: Confidence interval
CT: Computed tomography
CTCAE: Common Terminology Criteria for Adverse Events
HCC: Hepatocellular carcinoma
HR: Hazard ratio
IH-RFS: Intrahepatic recurrence-free survival rates
MLCs: Multileaf collimators
mRECIST: Modified Response Evaluation Criteria in Solid Tumors
OARs: Organs at risk
PEI: Percutaneous ethanol injection
RFA: Radiofrequency ablation
RILD: Radiation-induced liver disease
SBRT: Stereotactic body radiation therapy
TACE: Transarterial chemoembolization
VMAT: Volumetric-modulated arc therapy
